# Association between two mass-gathering outdoor events and incidence of SARS-CoV-2 infections during the fifth wave of COVID-19 in north-east Spain: A population-based control-matched analysis

**DOI:** 10.1016/j.lanepe.2022.100337

**Published:** 2022-02-28

**Authors:** Clara Suñer, Ermengol Coma, Dan Ouchi, Eduardo Hermosilla, Bàrbara Baro, Miquel Àngel Rodríguez-Arias, Jordi Puig, Bonaventura Clotet, Manuel Medina, Oriol Mitjà

**Affiliations:** a Fight AIDS and Infectious Diseases Foundation, Badalona, Badalona, Spain; b Sistemes d'Informació dels Serveis d'Atenció Primària (SISAP), Institut Català de la Salut (ICS), Gran Via de Les Corts Catalanes, 587, 08007, Barcelona, Spain; c IDIAP Jordi Gol, Universitat Autonoma de Barcelona, Barcelona, Spain; d ISGlobal, Hospital Clínic, Universitat de Barcelona, Barcelona, Spain; e Hospital Universitari Germans Trias i Pujol, Badalona, Spain; f Universitat de Vic-Universitat Central de Catalunya (UVIC-UCC), Vic, Spain; g Institut de Recerca de La Sida, IrsiCaixa AIDS Research Institute, Badalona, Spain; h Lihir Medical Centre- InternationalSOS, Lihir Island, Papua New Guinea

## Abstract

**Background:**

Many countries have resumed mass-gathering events like music festivals, despite the risk of severe acute respiratory syndrome coronavirus 2 (SARS-CoV-2) spreading. In this study, we aimed to assess the effect of two mass-gathering outdoor events, held during a peak of SARS-CoV-2 transmission, on COVID-19 incidence.

**Methods:**

This was a retrospective, population-based control-matched analysis. The study population included attendees to two outdoor music festivals held in Catalonia (North-East Spain). The primary objective was to compare the incidence of COVID-19 within the 3-to-10 days following the event between attendees and a population-based control group.

**Findings:**

The analysis included 18,275 and 27,347 attendees to the first and second festivals, respectively, and their corresponding controls. The post-festival 7-day cumulative COVID-19 incidence among attendees and controls was 4.14% (95% CI 3.86-4.44) vs. 1.69% (1.51-1.88) for the first festival (RR 2.46; 2.16-2.80), and 2.42% (2.35-2.61) and 1.10% (0.99-1.2) for the second festival (RR 2.19; 1.92-2.51). COVID-19 incidence among immunized individuals was also two-fold higher in attendees than in controls. Previous COVID-19 infection, vaccination, and adequate mask-wearing were significantly associated with a lower risk of COVID-19 infection after the events.

**Interpretation:**

Despite the proven effectiveness of preventive measures such as Ag-RDT screening, mask-wearing and vaccination, caution should be taken when holding these events during a period of high community SARS-CoV-2 transmission.

**Funding:**

Crowdfunding campaign YoMeCorono (https://www.yomecorono.com/) and the Generalitat de Catalunya.

**Research in context**

**Evidence before this study**

We searched PubMed for studies exploring the spreading of COVID-19 in outdoors mass-gathering events with preventive COVID-19 measures. The search was performed on Aug 1, 2021, and included the keywords “COVID-19”, “mass-gathering”, “RDT-screening” and “mask-wearing” with no language restriction. We found studies providing evidence of the low risk of holding these events in low transmission settings, while other authors reported an increase in COVID-19 cases associated to a spreading event where no measures had been implemented. Some authors investigated the risk of SARS-CoV-2 transmission using simulation studies. We found no published works specifically investigating the contribution of mass-gathering events to COVID-19 held during a peak of high SARS-CoV-2 transmission with preventives measures like Ag-RDT screening, mask wearing and vaccination.

**Added value of this study**

This is the first analysis to report that, during a peak of community transmission, the COVID-19 incidence among attendees to two music outdoor festivals (18,275 and 27,347 individuals, respectively) was two-fold higher than that of a population-based control group. The post-festival incidence of new cases per 100 persons-in-7-days among attendees and controls was 4.14 and 1.69 in the first festival, and 2.42 and 1.10 in the second one. Previous COVID-19 infection, vaccination, and adequate mask-wearing significantly reduced the risk.

**Implications of all the available evidence**

Mass-gathering events may impact COVID-19 incidence, like other social activities, when held during high community transmission of SARS-CoV-2. Our results provide policymakers with information to make evidence-based decisions on these types of events and on the protection conferred by vaccines, Ag-RDT screening and mask-wearing.

Alt-text: Unlabelled box

## Introduction

The high risk of spreading the severe acute respiratory syndrome coronavirus 2 (SARS-CoV-2) associated with mass-gathering events has encouraged the banning of many cultural and sporting events during the coronavirus disease 2019 (COVID-19) pandemic.^1^ While necessary for the public health control of COVID-19, the unprecedented closure of the entertainment industry has caused a remarkable impact on many economies and society wellbeing worldwide.^2^

In the past year, a deeper understanding of SARS-CoV-2 transmission dynamics has provided various measures for limiting virus transmission in these events.^3^ Of them, mask-wearing and point-of-care screening with antigen-detecting rapid diagnostic tests (Ag-RDT) have emerged as essential tools for creating a temporal safe environment during circumstances in which physical distance cannot be warranted.^4^, ^5^, ^6^, ^7^ When applied adequately, these preventive measures have shown the potential capacity to prevent SARS-CoV-2 spread, even in mass-gathering indoor events in a pre-vaccination scenario.^8^, ^9^, ^10^

Since the emergence of preliminary evidence on the effectiveness of these measures for creating safe environments, various mass-gathering events have been held, mostly during low transmission periods. However, their epidemiological impact has not been adequately assessed, leaving policymakers with limited information for making evidence-based decisions on these types of events in the rapidly evolving scenario of the COVID-19 pandemic. Besides organizational differences in the deployment of preventive measures, important unknowns exist that are key for the success of these preventive strategies. First, the negative predictive value of Ag-RDT drops with increasing local incidence of SARS-CoV-2 infection,^6^ potentially affecting the performance of Ag-RDT-based screenings. Second, preliminary evidence on this strategy was gathered before the emergence of the delta variant of SARS-CoV-2, with significantly higher transmissibility than early circulating variants and currently dominant in many countries worldwide.^11^

In this study, we retrospectively assessed the COVID-19 incidence associated with two large music festivals (capacity for over 10,000 people), which applied similar prevention strategies based on point-of-care Ag-RDT screening and mask-wearing, and were held during the fifth wave of transmission - predominantly caused by the delta SARS-CoV-2 variant - in Catalonia (North-East Spain).^12^

## Methods

### Study design and data sources

**Figure 1:**
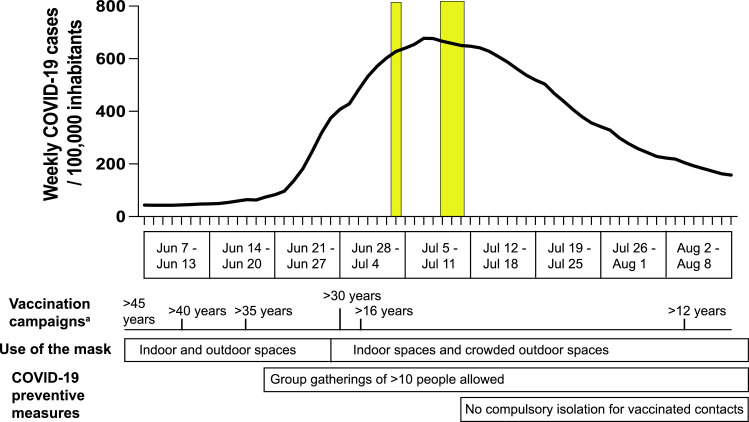
**Progression of cumulative weekly cases of COVID-19 per 100,000 inhabitants before and after the events in Catalonia.** Event dates are indicated with yellow bands; the first festival was held on 03 July and the second from 08 July to 10 July, 2021. The progressive role out of vaccination campaigns to younger individuals is indicated. Selected policies regarding the use of the face mask and other COVID-19 preventive measures are indicated. Incidence data was obtained from the public repository https://dadescovid.cat. ^a^ The time points indicate only the starting date of vaccination campaigns for each age group. Healthcare and other essential workers of all ages were vaccinated during the first semester of 2021, before the indicated date for their corresponding age group.

This was a retrospective control-matched analysis of COVID-19 incidence after two mass-gathering outdoor events, both music festivals, in Barcelona (Catalonia, North-East Spain). The first event was held on July 3, 2021, and second on July 8-10, 2021. By the time of the events, Catalonia was experiencing the fifth wave of the COVID-19 outbreak, and the weekly incidence reached 669 cases per 100,000 inhabitants on July 6-12, 2021 (Figure 1). Public health directives and vaccination campaigns in force at that time are included in Figure 1.

Our analysis included all the individuals who attended any of the events with demographic and COVID-19-related information (i.e., sex, age, previous PCR or Ag-RDT results, and vaccine doses received) available in the central health registry of the Catalan Health Service. This registry contains essential demographic and clinical data from nearly 7.7 million people and has been used for relevant COVID-19 research previously.^13^, ^14^, ^15^ The same central health registry was used to build a matched population-based control group for each attendant of the events. Attendees were paired 1:1 by exact matching to individuals of the general population according to sex, age (+/- 2 years), healthcare area of residence, vaccination status, and history of a positive result in Ag-RDT or PCR. Supplementary methods on the case-control study are provided in the Supplementary Appendix 1.

The second festival included a survey-based assessment of risk behaviours during the event. The survey, which was answered anonymously online, was sent to all individuals who attended the second festival 14 days after ending the event, and included items such as the exhaustivity in the use of face masks and the overall fulfilment of the preventive measures (Supplementary Appendix 2). Attendees had five days to answer the survey, and the organizers raffled festival ticket prizes among survey participants as an incentive.

The study protocol for secondary use of healthcare data and administration of a post-event survey was approved by the independent ethics committee of the University Hospital Germans Trias i Pujol in Badalona (Spain). All data were handled according to the General Data Protection Regulation 2016/679 on data protection and privacy for all individuals within the European Union and the local regulatory framework regarding data protection.

### Events characteristics and preventive measures

Both events were commercial music festivals with pre-purchase entrance system. The first festival was a one-day event (Saturday from 2:00 pm to 6:30 am) held in a 33,000 m^2^ area with capacity for 25,000 people. The second festival was a 3-day event (Thursday from 4:00 p.m. to 1:00 a.m., Friday and Saturday from 4:00 p.m. to 5:00 a.m.), celebrated in a 100,351 m^2^ area with a maximum capacity for 25,000 people.

The two events followed a similar approach for preventing SARS-CoV-2 spread, based on face mask use, forehead temperature measurement, and Ag-RDT screening of nasopharyngeal swab for SARS-CoV-2. In both festivals, attendees were provided with an FFP2 mask, which they could renew during the event if needed. The use of the mask was compulsory, except for eating or drinking. Eating and drinking was allowed in all areas at the first festival. At the second festival, eating and drinking was not allowed in the area in front of the stage, where organization staff oversaw the event and warned people not wearing the mask. Attendees were allowed to move around without restrictions and no social distancing measures were in place. Regarding Ag-RDT screening, attendees to both festivals had to make an appointment for the same day of the entrance and pay for the Ag-RDT, performed by trained staff. However, the two festivals differed in the organization of Ag-RDT screening (Supplementary Appendix 1). Table S1 (Supplementary appendix 1) provides an estimate of the expected negative predictive value associated with Ag-RDT screening for a range of local prevalence values (i.e., pre-test probabilities).

### Study outcomes

The primary outcome was the post-event cumulated incidence of SARS-CoV-2 infections confirmed by a diagnostic test (PCR or Ag-RDT). The incidence was calculated using the central health registry of the Catalan Health Service. Because SARS-CoV-2 is a mandatory notifiable infection to all public and private laboratories, the register is comprehensive and captures all diagnostics of SARS-CoV-2 in patients with symptoms, contact-tracing, or any other reasons for testing. Based on the high COVID-19 incidence and dominance of the delta variant in the area during the event, we constrained the time frame for the primary analysis to 3-to-10 days after starting the event, to maximize the likelihood of the transmission to have occurred during the event. Covid-19 incidence within different post-event periods is shown in Table S3 (Supplementary Appendix 1).

Secondary outcomes included the cumulated incidence according to the immunity status, which was stratified into the following categories: fully protected (i.e., had received the complete vaccination regimen or one vaccine dose among individuals with a history of natural SARS-CoV-2 infection, more than 14 days before attending the festival), partially protected (i.e., either a history of SARS-CoV-2 infection without a vaccine, one dose of a two-dose regimen vaccine, or having received a complete vaccination regimen within the 14 days before attending the festival), and unprotected (i.e., unvaccinated with no evidence of previous SARS-CoV-2 infection). Based on the local guidelines in force by the time of the events, we considered the "complete vaccination regimen" for all individuals who had received two vaccine doses of Pfizer/BioNTech, Moderna/Lonza, and Oxford/AstraZeneca vaccines, or one dose for J&J/Janssen, at least 14 days before the event.

### Risk factor analysis

For the analysis of risk factors for COVID-19 infection after the event, the time frame considered comprised the 3-to-10 days after the event or the first day of attendance in the case of the survey. Attendees that did not report a COVID-19 infection during this time frame were used as controls. For the survey analysis, cases reported outside this time frame were excluded from the analysis. As potential risk factors, when analysing the data from the electronic health records, the following variables were considered: sex, age, history of previous COVID-19 infection, and being partially or fully vaccinated. Full vaccination comprised individuals who had received a complete vaccination regimen >14 days before the event, and partial vaccination comprised individuals who had received an incomplete vaccination regimen or a complete vaccination regimen <14 days before the event. In the subset of attendees who responded to the post-event survey after the second event, the incidence was also stratified according to the number of days attending the event (1 to 3), and self-reported behaviour factors, including mask wearing all or most of the time (5-point qualitative scale).

### Statistical analysis

Categorical and quantitative variables were described as frequency and percentages and mean and standard deviation (SD) over available data, respectively. The cumulative incidence of COVID-19 infections per hundred people observed within 3 to 10 days after the event was compared between the attendees of each festival and its matched population-based controls using the relative risk (RR) and its 95% confidence interval (CI). No data imputation was made. The influence of baseline and behaviour factors was investigated using a logistic regression model for reporting a positive COVID-19 result within 3 to 10 days following the event, based on the electronic health records and survey-collected data. The model included age, gender, vaccination level, and previous infection record in each of the two study populations: the overall sample of attendees for each event and the sample of respondents to the post-event survey. In addition, for the post-event survey sample, we added the use of the mask, the degree of compliance with protection measures, and the number of days attending the event. Assuming a possible interaction between variables, we performed pairwise Pearson correlation and compared nested models with the F-test. All analyses were performed in R v4.1.0.^16^

## Results

### Characteristics of the study population

According to the organizers, the first festival identified 152 individuals with a positive result in the Ag-RDT screening and allowed the entrance of 21,012 individuals with a negative result. The number of individuals with a positive Ag-RDT (excluded) and total attendees in the second festival was 292 and 34,518 individuals, respectively. Of these, the second festival received 11,134; 16,655 and 15,650 attendees Thursday, Friday and Saturday, respectively. The two study samples, including attendees with enough data for control-matched pairing with the general population, consisted of 18,275 (87.0% of total attendees) and 27,347 (79.2% of total attendees) for the first and second festival, respectively (Figure S1, Supplementary appendix 1).

Table 1 summarizes the demographic and COVID-19 immunity status of the individuals included in the analysis. The first event had a lower mean age (25.5 (SD 9.4) vs 33.0 (SD 8.8) years), and a higher percentage of females (11,923 (65.2%) vs. 14,236 (52.1%) women) than the second. Regarding the previous SARS-CoV-2 infections, 1,737 (9.5%) and 2,862 (10.5%) of the attendees had a history of COVID-19 confirmed diagnosis (either PCR or Ag-RDT), in the first and second event, respectively. The immunity status of attendees in the first and second event, respectively, was as follows: 3,672 (20.1%) and 6,373 (23.3%) were fully protected (i.e., full vaccination regimen or one vaccine dose and a history of COVID-19), 4,280 (23.4%) and 11,991 (43.8%) were partially protected (i.e., one dose of a two-dose vaccine regimen or two doses <14 days before the event, or non-vaccinated with COVID-19 history), and 10,323 (56.5%) and 8,983 (32.8%) were unprotected (i.e., without any vaccine dose and no history of COVID-19). Among vaccinated individuals, partially vaccinated individuals had received their last vaccine dose within less days than fully vaccinated individual (time since last vaccine dose in days [Median (IQR)], partially vaccinated vs fully vaccinated attendees: 12 [2-25] vs 33 (27-114), and 11 [6-22] vs 40 (30-142), for the first and second festivals, respectively). The characteristics of the attendees that were not included because no control-match pairing was found are summarized in Table S2 (Supplementary Appendix 1).

**Table 1:** Main characteristics of individuals included in the two investigated cohorts: population-based analysis (individuals who attended the event and had enough data in central healthcare registries for a population-based matched control group) and survey (individuals who responded the 14-day post-event survey after the second festival).

	Population-based analysis		Survey Second Festival (N=13,137)
	First Festival (N =18,275)	Second Festival (N =27,347)	
**Age (SD)**	25.5 (9.43)	33.0 (8.84)	34.6 (8.54)
**Sex/Gender**^a^			
Female	11,923 (65.2%)	14,236 (52.1%)	7,582 (57.7%)
Male	6,352 (34.8%)	13,111 (47.9%)	5,471 (41.6%)
Non-binary	-	-	84 (0.6%)
**Previous COVID-19 Infection**			
No	16,538 (90.5%)	24,485 (89.5%)	11,520 (87.7%)
Yes	1,737 (9.50%)	2,862 (10.5%)	1,617 (12.3%)
**COVID-19 immunity Status**^b^			
Unprotected	10,323 (56.5%)	8,983 (32.8%)	2,996 (22.8%)
Partially Protected	4,280 (23.4%)	11,991 (43.8%)	5,554 (42.3%)
Fully Protected	3,672 (20.1%)	6,373 (23.3%)	4,587 (34.9%)

a For Population-based analysis, sex was obtained from central health registries. For Survey analysis, gender was self-reported.

b Categories of the COVID-19 immunity status were as follows: fully protected (i.e., had received the complete vaccination regimen or one vaccine dose among individuals with a history of natural SARS-CoV-2 infection), partially protected (i.e., either a history of SARS-CoV-2 infection without a vaccine, one dose of a two-dose regimen vaccine, or a complete vaccination regimen <14 days before the event), and unprotected (i.e., unvaccinated with no evidence of previous SARS-CoV-2 infection). SD: Standard deviation.

Of the 34,518 attendees of the second event, 13,137 (38.1%) responded to the survey sent 14 days after the event. The mean age (34.6 years, SD 8.5) and the proportion of females (57.7%) were slightly higher in survey respondents compared to the overall cohort population (Table 1, Table S2). Individuals who attended the event the first day responded less frequently than those who attended the other two days (response rates for Thursday, Friday and Saturday attendees were 22.4%, 40.7% and 37.1%, respectively). The highest response rate was observed among individuals attending the event the three days (54.5%). Regarding the immunity status of survey respondents, 4,587 (34.9%) were fully protected, 5,554 (42.3%) partially protected, and 2,996 (22.8%) unprotected. The time since last vaccine dose in days [Median (IQR)], for partially vaccinated and fully vaccinated survey responders was 37 (8-38) and 39 (37-129), respectively.

### COVID-19 incidence

The number of attendees who had a positive test for SARS-CoV-2 reported to the central health registry of the Catalan Health System within the 3 to 10 days following the event was 757 (4.14%; 95% CI 3.86% - 4.44%) and 662 (2.42%; 2.32% - 2.61%) for the first and the second festival, respectively. The risk ratio (RR) for reporting a COVID-19 case within the 3-10 days following the event regards to the population-based control group was 2.46 (2.16 - 2.80) and 2.19 (1.92 - 2.51) for the first and second festivals, respectively (Table 2). The RR for a COVID-19 case was similar, regardless of the immunization status, although incidence was significantly higher in non-immunized individuals among both, attendees and population-based control individuals (Table 2). Table S3 (Supplementary appendix 1) summarizes the incidences and risk estimated for different post-event periods. The overall RR for COVID-19 within the first 3 days after the events were 0.74 (0.53 - 1.03) and 0.21 (0.14 - 0.31) for the first event and second festivals, respectively (Table S3).

**Table 2:** Incidence of COVID-19 3-to-10 days after the events and relative risk of attendees compared with a matched population-based control group.

		Attendees			Controls			Relative Risk	95% CI	
	Cohort	COVID-19 Cases	%	95% CI	COVID-19 Cases	%	95% CI			
FIRST FESTIVAL										
**All attendees**	18,275	757	4.14%	(3.86% - 4.44%)	308	1.69%	(1.51% - 1.88%)	2.46	(2.16 - 2.80)	
**According to immunity status**										
Fully protected	3,672	67	1.82%	(1.44% - 2.31%)	31	0.84%	(0.60% - 1.20%)	2.16	(1.42 - 3.30)	
Partially protected	4,280	79	1.85%	(1.48% - 2.29%)	39	0.91%	(0.67% - 1.24%)	2.03	(1.38 - 2.97)	
Unprotected	10,323	611	5.92%	(5.48% - 6.39%)	238	2.31%	(2.03% - 2.61%)	2.57	(2.22 - 2.97)	
SECOND FESTIVAL										
**All attendees**	27,347	662	2.42%	(2.35% - 2.61%)	302	1.10%	(0.99% - 1.2%)	2.19	(1.92 -2.51)	
**According to immunity status**										
Fully protected	6,373	96	1.51%	(1.24% - 1.84%)	42	0.66%	(0.49% - 0.89%)	2.29	(1.6 - 3.32)	
Partially protected	11,991	182	1.52%	(1.31% - 1.75%)	112	0.93%	(0.78% - 1.12%)	1.63	(1.3 - 2.05)	
Unprotected	8,983	384	4.27%	(3.88% - 4.71%)	148	1.65%	(1.4% - 1.93%)	2.59	(2.15 - 3.13)	

^a^Immunity status categories: fully protected (i.e., had received the full vaccination regimen or one vaccine dose among individuals with history of natural SARS-CoV-2 infection), partially protected (i.e., either a history of SARS-CoV-2 infection without vaccine, an incomplete regimen of vaccination, or a complete vaccination regimen <14 days before the event), and unprotected (i.e., unvaccinated with no evidence of previous SARS-CoV-2 infection). **CI:** confidence interval. **RR:** relative risk.

### Risk factors for COVID-19 during the events

**Figure 2:**
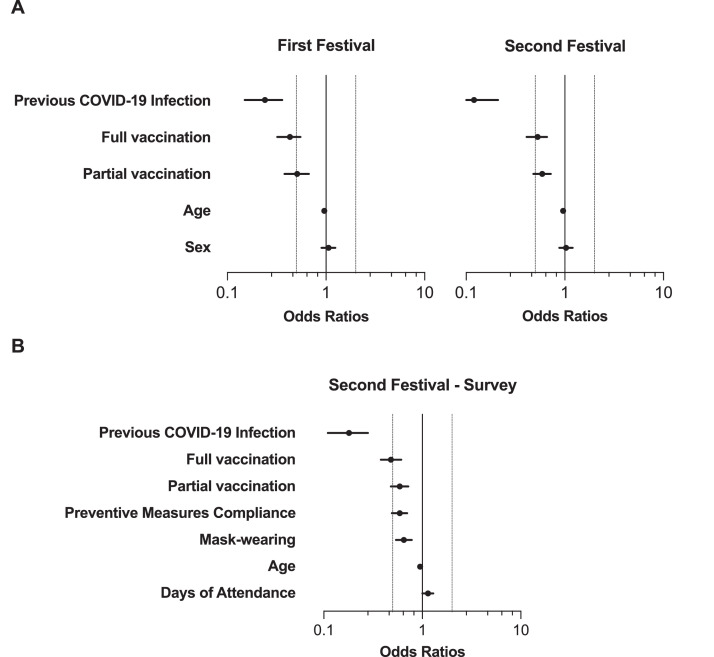
**Odds ratio for COVID-19 infection after attending the events.** Risk factor multivariate analysis for COVID-19 infection after attending the events. Time frame considered: 3-to-10 days. (**A)** Analysis including all attendees and variables recorded in the central health registries [n(cases)/n(total): 757/18,275 for the first festival; 662/27,347 for the second festival). (**B)** Sub analysis of attendees who responded the 14-day survey regarding behavioural factors after attending the second festival [n(cases)/n(total): 570/13,097]. Definitions: Previous COVID-19 Infection (with or without receiving any vaccine); Full vaccination (i.e., had received the full vaccination regimen >14 days before the event), partial vaccination (i.e., had received an incomplete vaccination regimen or a complete vaccination regimen <14 days before the event); Preventive Measures Compliance and Mask-wearing (OR for answering "all or most of the time"); Age (OR for an increase in one unit); Days of Attendance (OR for each extra day of assistance). Dashed vertical lines indicate Odds Ratios of 0.5 and 2. Alternative models including the interaction between age and gender showed no contributions to the model (ORs of the interaction age*gender were 0.99 [0.96-1.01] for the first festival, 1.00 [0.98-1.02] for the second, and 1.00 [0.98-1.02] for the survey). The values for OR and 95% CI are presented in Table S4.

The logistic regression analyses for the two events consistently identified age, vaccination (either partial or full regimen), and history of COVID-19 as contributors to reducing the likelihood of experiencing a COVID-19 episode within the 3-10 days following the event (Figure 2a, Table S4). The effect size of each of the factors was similar in the two events, with a history of previous COVID-19 being associated with the lowest in either of the events.

The anonymous survey launched 14 days after the second event was answered by 13,137 individuals. Of them, 570 (4.34%) reported having been diagnosed of COVID-19 case to the central health registry within the 3-10 days following the event. The incidence was lower among fully protected individuals (118/4,587; 2.57%) and fully protected individuals who stated they had used the face mask all the time (67/3,452; 1.94%).

The multivariate analysis among survey respondents confirmed age and immunity (either complete or partial, natural or vaccine-driven) as factors with significant contribution to reducing the odds of COVID-19 (Figure 2b, Table S4). Additionally, behavioural factors such as complying with the overall preventive measures and mask-wearing (all the time or most of the time, corresponding to the two highest scores on the 5-point scale) also reduced the odds of COVID-19. Although the variables “mask-wearing” and “compliance with the overall preventive measures” showed a positive correlation (rho=0.23), the nested version excluding any of the two variables did not improve the model fit (Figure S2). Another variable pair with important correlation included the vaccination status and age (rho=0.35), presumably due to the age-driven rollout of vaccination campaigns (Figure 1). Each additional attended day significantly increased risk of experiencing COVID-19 by 1.14. Considering that all participants attended the event at least one day, a RR of 1.14 and 1.30 were associated with attending the festival two days (i.e., one additional day) and the entire festival (i.e., two additional days), respectively.

## Discussion

Attendance to an outdoors mass-gathering event held during a COVID-19 transmission peak was independently associated with a two-fold risk of post-event COVID-19 compared to a population-based matched control group of non-attendees. These findings were consistent in two independent music festivals with a similar approach for preventing SARS-CoV-2 spread, which included compulsory mask-wearing and point-of-care screening with Ag-RDT. We also found that previous COVID-19 infection, vaccination, adequate mask-wearing, and following general recommendations for preventing SARS-CoV-2 spread were significantly associated with a lower risk of COVID-19. However, COVID-19 incidence among immunized festival attendees was also two-fold higher than controls.

The increased risk observed in our cohort of attendees was in line with that reported for other social activities, such as going to restaurants (OR 1.95 - 2.8) and bars (OR 1.95 - 3.9).^17^, ^18^, ^19^, ^20^ The effectiveness of face mask-wearing and Ag-RDT testing for preventing SARS-CoV-2 spread in this type of events had been suggested in previous experiences, which could not show an increased risk of SARS-CoV-2 transmission.^8^, ^9^, ^10^ However, these previous studies were conducted during periods of low COVID-19 incidence in the background population, thus resulting in an overall limited number of cases. The festivals included in our study were held in a moment of notably higher background incidence in the area: 620-to-658 7-day cumulative COVID-19 incidence per 100,000 inhabitants by the time of the outdoor events investigated vs. the 96-to-132 range reported in previous assessments of the impact of mass-gathering indoor events.^8^, ^9^, ^10^ The higher transmission in the outdoor events included in our study highlights the relevance of the background COVID-19 incidence regarding the safety of mass-gathering events, which could be compromised by the sharp increase on the number of false negative results of Ag-RDTs with increasing local prevalence (i.e., pre-test probability) (Table S1, Supplementary Appendix 1).^21^

One of the benefits from the Ag-RDT screening strategy was the identification of a remarkable number of asymptomatic SARS-CoV-2-infected individuals, which was close to the absolute excess cases in one of the festivals. The screening effectiveness was clearly manifested by a lower risk of experiencing COVID-19 within the first 3 days following the event among attendees compared controls. Therefore, the identification and possibility of contact-tracing and quarantine of these cases that would be otherwise unnoticed should also be considered when appraising the overall impact of these events in the pandemic course. Likewise, other factors such as clinical severity (likely associated with the individual profile), psychological, social, and economic wellbeing of these events should be considered.

Our population-based analysis is strengthened by the analytical approach and the building of a population-based control group matched by age, sex, healthcare area of residence and immunity status for SARS-CoV-2. Although positive experiences with these types of events have been conducted elsewhere, incidence data after the event is often reported in press releases, with little certainty of accuracy,^22^, ^23^, ^24^ or descriptive studies without formal comparison with non-attendees that allow estimating the actual risk associated with the event.^9^^,^^25^ On the one hand, the population-based control-matched approach constrained our study sample to individuals with available data on the vaccination status and basic demographic information in administrative health records, warranting high exhaustivity and representativeness of the datasets. Although the high number of individuals included in the analysis (45,622 overall) provides confidence in our risk estimates, this approach limited the analysis sample, which excluded attendees without information in the central Healthcare Registry (presumably non-Catalan individuals) and those without individuals in the general population meeting the matching criteria. Another limitation of our population-based analysis was the unfeasibility of considering behavioural or socioeconomic characteristics for the pairing. Although the immunity status is expected to be the most relevant factor for COVID-19, virus transmission may also be influenced by social behaviours, health awareness, fulfilling of public health recommendations, and factors related to the socioeconomic status, including the place of residence, profession, or household occupation.^26^^,^^27^

Besides the population-based control-matched analysis, we collected behavioural information in one of the two investigated events. The post-event survey showed that wearing a face mask and complying with protective measures most of the time was independently associated with a lower risk of COVID-19. Attending two or three days to a multiday festival also increased the risk of COVID-19 by 14% and 30%, respectively. The results from the post-event anonymous survey must be read in the context of the limited and not randomized sample of respondents, who were biased towards older-, female- and 3-days attendees. The positivity rate among survey responders also indicated that positive cases were more likely to answer. Thus, results from this analysis could be affected by these confounding factors. Furthermore, we cannot rule out the recall bias (i.e., inaccurate recollection of events by respondents) and social desirability bias (i.e., a tendency to give a favourable view of one's self).^28^ Nevertheless, anonymity, the high number of respondents (over 13,000 individuals), and the consistency of the results with previous evidence regarding the use of masks in crowded outdoor environments^29^ support the importance of this measure in preventing SARS-CoV-2 spread in mass-gathering events.

In summary, our results raise awareness on the limitations of Ag-RDT screening and mask-wearing for containing SARS-CoV-2 transmission in mass-gathering events held during high COVID-19 incidence. Although COVID-19 incidence was lower among vaccinated attendees, the higher risk compared with controls was maintained, supporting the use of additional measures besides vaccination certificates. Therefore, these findings are relevant for countries with high vaccination rates that are experiencing an increase of COVID-19 incidence and ―more importantly― low- and middle-income countries with low vaccination rates, where the pandemic still wreaks havoc.

Availability of data sources: Data that support the findings of this study are openly available on the Supplementary Appendix 3.

## Declaration of interests

The authors declare no competing interests.

## Appendix Supplementary materials

Image, application 1

Image, application 2

Image, application 3

Image, application 4

Image, application 5

Image, application 6
