# Supplementary material for: Association between two mass-gathering outdoor events and incidence of SARS-CoV-2 infections during the fifth wave of COVID-19 in north-east Spain: A population-based control-matched analysis
Source: Lancet Reg Health Eur. 2022 Feb 28;15:100337. doi: 10.1016/j.lanepe.2022.100337 (PMC8883024; doi:10.1016/j.lanepe.2022.100337)
Supplement: Supplementary file 1 [file mmc1.pdf]

Association between Two Mass-Gathering Outdoor Events and Incidence of SARS-CoV-2 Infections during the Fifth Wave of COVID-19 in North-East Spain: a Population-Based Control-Matched Analysis

Supplementary Appendix 1

Content:

1. Supplementary material captions

2. Supplementary methods

3. Table S1

4. Figure S1

5. Table S2

6. Table S3

7. Table S4

8. Figure S2

Supplementary materials Captions

Table S1. Estimate of the number of false negative results according to attendees in each festival, two test sensitivity values, and a range of pre-test probabilities. The number of false negative (FN) tests was derived from the negative-predictive value (NPV), estimated by considering the prevalence as pre-test probability. Several NPV were modeled for pre-test probabilities ranging between 0.01% to 2% given an expected specificity of 95% and two sensibilities: 70 and 80%. Estimates were modeled separately for the number of attendees in each event and two different sensitivity values.

Figure S1. Study Profile. Flow-chart of individual inclusion in the analysis. Flowchart for the first (A) and second (B) festival outdoor music festivals.

Table S2. Population characteristics. Demographic characteristics for individuals included in the analysis. SARS-CoV-2 immunity status is specified.

Table S3. Covid-19 Incidence and relative risk (RR) within different post-event periods

Table S4. Odds ratio for COVID-19 infection after attending the events (Numerical values). Risk factor multivariate analysis for COVID-19 infection after attending the events. Time frame considered: 3-to-10 days. Definitions: Previous COVID-19 Infection (with or without receiving any vaccine); Full vaccination (i.e., had received the full vaccination regimen >14 days before the event), partial vaccination (i.e., had received an incomplete vaccination regimen or a complete vaccination regimen <14 days before the event); Preventive Measures Compliance and Mask-wearing (OR for answering "all or most of the time"); Age (OR for an increase in one unit); Days of Attendance (OR for each extra day of assistance). These numerical values are represented as a forest plot in Figure 2.

Figure S2. Correlation analysis between risk factors. (A) Pearson correlation between risk factors. Coefficients with p values < 0.01 are shown. **(B)** Residual deviance of the logistic regression model including the variables “mask-wearing” and “compliance with the overall preventive measures (Figure 2B of the main text) and excluding the item regarding “compliance with preventive measures” were 4330 and 4365, respectively (F-statistic 34.98; p <.001).

1. Supplementary methods

Case-control study

The organizers of each festival collected demographics of the attendees and the Ag-RDT result at the entrance of each festival. The same organizers also collected the individuals consent to capture and use this data for epidemiological and research purposes. After the events, data identifying festival attendees was transferred to the Catalan Health Authorities. Researchers at the Catalan Health Department collected information regarding the Covid-19 vaccination status of the attendees and paired each individual to a control subject (adjusting for age, gender, basic health area and Covid-19 immune status). Finally, data about positive SARS-CoV-2 test of the attendees and the controls within the next days after the festival was collected from the Catalan Health Department registry. This data was transferred anonymously (without any personal identificatory) to investigators from the Germans Trias i Pujol Hospital and Lluita contra la Sida Foundation to be interpreted. Supplementary Figure 1A and 1B show a diagram of the study profile for the first and second festival, respectively. Available demographic data are shown in Supplementary Table 2.

Ag-RDT screening organization in each Festival

The first festival set multiple testing points across the country; individuals who tested negative were provided with a new ticket that allowed them to access the festival. The second festival provided all registered individuals with an electronic bracelet linked to their personal profile and aimed to give them access to the festival and make contactless payments during the event. Each day before entering the event, individuals underwent Ag-RDT at the venue entrance. Approximately 15 minutes after the test, the result was notified by email and recorded in the bracelet profile; individuals with a negative result were allowed to enter the event.

Table S1. Estimate of the number of false negative results according to attendees in each festival, two test sensitivity values, and a range of pre-test probabilities.

Event	Sensitivity and Specificity	Pre-test probability	Negative Predictive Value	True Negatives [n (%)]	False Negatives [n (%)]
		2%	0·996	20,571 (97·9%)	84 (0·4%)
**FIRST**	Sensitivity 0·8	1%	0·998	20,781 (98·9%)	42 (0·2%)
**FESTIVAL**		0.5%	0·999	20,886 (99·4%)	21 (0·1%)
Number of	Specificity 0·999	0.1%	1·000	20,970 (99·8%)	4 (0·0%)
Attendees:		0.01%	1·000	20,989 (99·9%)	0 (0·0%)
21,012		2%	0·994	20,571 (97·9%)	126 (0·6%)
	Sensitivity 0·7	1%	0·997	20,781 (98·9%)	63 (0·3%)
		0.5%	0·998	20,886 (99·4%)	32 (0·2%)
	Specificity 0·999	0.1%	1·000	20,970 (99·8%)	6 (0·0%)
		0.01%	1·000	20,989 (99·9%)	1 (0·0%)
		2%	0·996	33,794 (97·9%)	138 (0·4%)
**SECOND**	Sensitivity 0·8	1%	0·998	34,139 (98·9%)	69 (0·2%)
**FESTIVAL**		0.5%	0·999	34,311 (99·4%)	35 (0·1%)
Number of	Specificity 0·999	0.1%	1·000	34,449 (99·8%)	7 (0·0%)
Attendees:		0.01%	1·000	34,480 (99·9%)	1 (0·0%)
34,518		2%	0·994	33,794 (97·9%)	207 (0·6%)
	Sensitivity 0·7	1%	0·997	34,139 (98·9%)	104 (0·3%)
		0.5%	0·998	34,311 (99·4%)	52 (0·2%)
	Specificity 0·999	0.1%	1·000	34,449 (99·8%)	10 (0·0%)
		0.01%	1·000	34,480 (99·9%)	1 (0·0%)

Figure S1. Study Profile. Flow-chart of individual inclusion in the analysis.

A

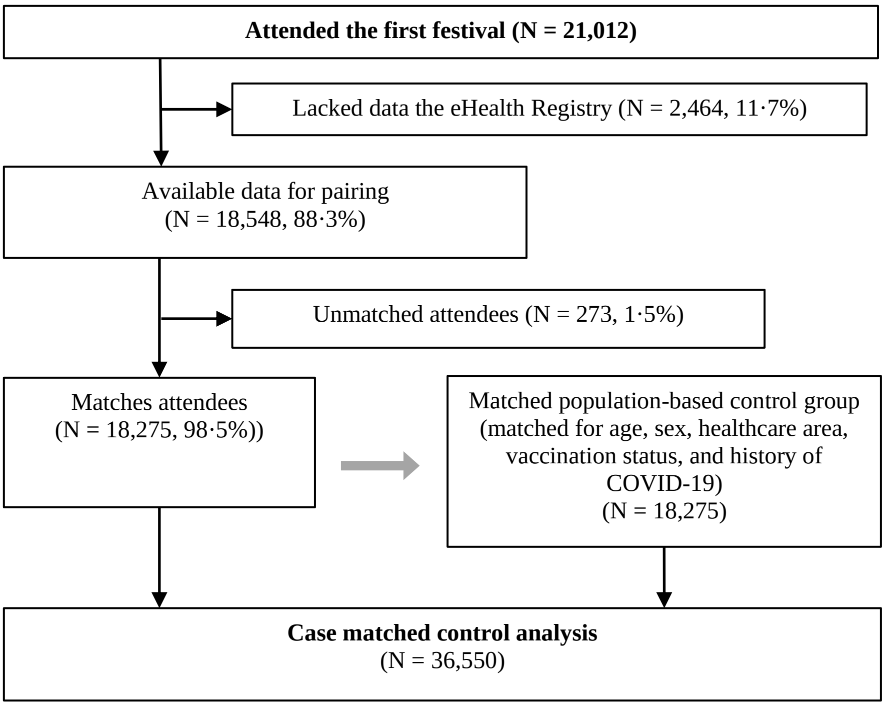

B

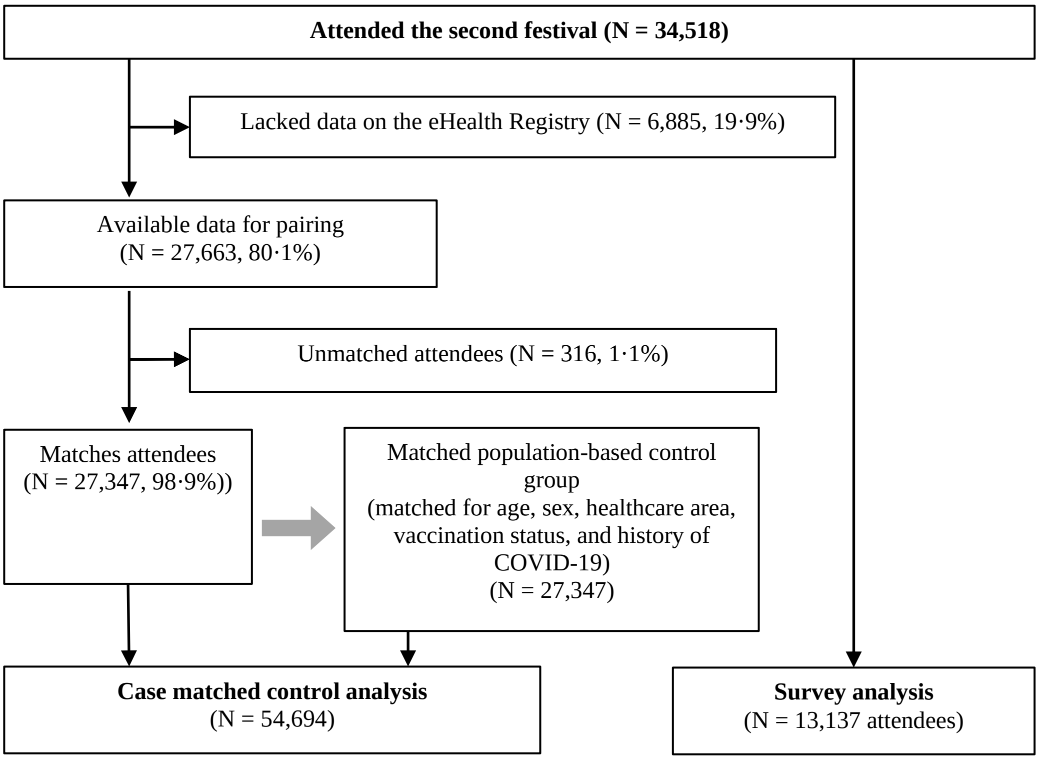

Table S2. Population characteristics

	First Festival			Second Festival			
	All attendees[a] (N = 21,012)	Attendees at the eHealth Registry[b] (N = 18,548)		All attendees[c] (N = 34,518)	Attendees at the eHealth Registry[b] (N = 27,663)		Survey[d] (N = 13,137)
		Population-based analysis (N =18,275)	Unmatched attendees (N =273)		Population-based analysis (N =27,347)	Unmatched attendees (N = 316)	
**Age (SD)**	-	25·5 (9·43)	26.0 (7.90)	33·0 (8·7)	33·0 (8·84)	30·2 (6·82)	34·6 (8·54)
**Sex/Gender**							
Female	-	11,923 (65·2%)	155 (56.8%)	17,841 (51·7%)	14,236 (52·1%)	130 (41·1%)	7,582 (57·7%)
Male	-	6,352 (34·8%)	118 (43.2%)	16,351 (47·4%)	13,111 (47·9%)	186 (58·9%)	5,471 (41·6%)
Non-binary	-	-	-	325 (0·9%)	-	-	84 (0·6%)
**Previous COVID-19 Infection**							
No	-	16,538 (90·5%)	32 (11.7%)	-	24,485 (89·5%)	35 (11·1%)	11,520 (87·7%)
Yes	-	1,737 (9·50%)	241 (88.3%)	-	2,862 (10·5%)	281 (88·9%)	1,617 (12·3%)
**COVID-19 immunity Status[e]**							
Unprotected	-	10,323 (56·5%)	75 (27.5%)	-	8,983 (32·8%)	65 (20·6%)	2,996 (22·8%)
Partially Protected	-	4,280 (23·4%)	33 (12.1%)	-	11,991 (43·8%)	60 (19·0%)	5,554 (42·3%)
Fully Protected	-	3,672 (20·1%)	165 (60.4%)	-	6,373 (23·3%)	191 (60·4%)	4,587 (34·9%)

[a] Demographics for all attendees at the first festival were not available.
[b] Data obtained from the electronic Health Registry. Only attendees that were found in this registry were included.
[c] Demographics for all attendees at the second festival were self-reported by the attendees to the festival organizers when the attendees scheduled their Ag-RDT test at the screening. To calculate the mean age, only ages between 0 and 99 years were considered (367 values were excluded).
[d] Demographics and Covid-19 immunity status were self-reported by the attendees that responded a survey after attending the second festival.
[e] Categories of the COVID-19 immunity status were as follows: fully protected (i.e., had received the complete vaccination regimen or one vaccine dose among individuals with a history of natural SARS-CoV-2 infection), partially protected (i.e., either a history of SARS-CoV-2 infection without a vaccine, one dose of a two-dose regimen vaccine or a complete vaccination regimen <14 days before the event), and unprotected (i.e., unvaccinated with no evidence of previous SARS-CoV-2 infection).

Table S3. Covid-19 Incidence and relative risk (RR) within different post-event periods

FIRST FESTIVAL

	**Attendees**			**Controls**				
	Cases	%	CI95	Cases	%	CI95	RR	IC95
0-14 days	987	5·40%	(5·08% - 5·74%)	534	2·92%	(2·69% - 3·18%)	1·85	(1·67 - 2·05)
< 3 days	59	0·32%	(0·25% - 0·42%)	80	0·44%	(0·35% - 0·54%)	0·74	(0·53 - 1·03)
1-10 days	813	4·45%	(4·16% - 4·76%)	366	2·00%	(1·81% - 2·22%)	2·22	(1·9 - 2·5)
**3-10 days**	**757**	**4·14%**	**(3·86% - 4·44%)**	**308**	**1·69%**	**(1·51% - 1·88%)**	**2·46**	**(2·16 - 2·8)**
3-6 days	531	2·91%	(2·67% - 3·16%)	183	1·00%	(0·87% - 1·16%)	2·90	(2·46 - 3·43)
3-14 days	928	5·08%	(4·77% - 5·41%)	454	2·48%	(2·27% - 2·72%)	2·04	(1·83 - 2·28)
> 14 days[a]	209	1·14%	(1·0% - 1·31%)	163	0·89%	(0·77% - 1·04%)	1·28	(1·04 - 1·5)

SECOND FESTIVAL

	**Attendees**			**Controls**				
	Cases	%	CI95	Cases	%	CI95	RR	IC95
0-14 days	875	3·20%	(3·0% - 3·41%)	583	2·13%	(1·97% - 2·31%)	1·50	(1·35 - 1·66)
< 3 days	27	0·10%	(0·07% - 0·14%)	131	0·48%	(0·40% - 0·57%)	0·21	(0·14 - 0·31)
1-10 days	683	2·50%	(2·32% - 2·69%)	373	1·36%	(1·23% - 1·51%)	1·83	(1·62 -2·1)
**3-10 days**	**662**	**2·42%**	**(2·35% - 2·61%)**	**302**	**1·10%**	**(0·99% - 1·2%)**	**2·19**	**(1·92 -2·51)**
3-6 days	411	1·50%	(1·37% - 1·65%)	186	0·68%	(0·59% - 0·78%)	2·21	(1·86 -2·62)
3-14 days	848	3·10%	(2·90% - 3·31%)	452	1·65%	(1·51% - 1·81%)	1·88	(1·67 -2·1)
> 14 days [a]	142	0·52%	(0·44% - 0·61%)	141	0·52%	(0·44% - 0·61%)	1·01	(0·8 - 1·27)

[a] >14 days: 14-24 days post-event

CI: confidence interval

RR: relative risk

Table S4. Numerical values for multivariate model for COVID-19 infection after attending the events.

	eHealth Records				Survey	
	First Festival		Second Festival		Second Festival	
**Predictors**	**OR**	**CI**	**OR**	**CI**	**OR**	**CI**
Age	0.96	0.95 - 0.97	0.96	0.95 - 0.97	0.95	0.94 - 0.96
Gender	1.06	0.90 - 1.24	1.03	0.88 - 1.20	0.97	0.81 - 1.16
Half Vaccination	0.51	0.38 - 0.67	0.59	0.48 - 0.72	0.58	0.47 - 0.72
Complete Vaccination	0.43	0.32 - 0.55	0.53	0.41 - 0.66	0.48	0.38 - 0.61
Previous Covid-19 Infection	0.24	0.15 - 0.36	0.12	0.06 - 0.20	0.18	0.11 - 0.28
Days of Attendance	-	-	-	-	1.14	1.00 - 1.29
Mask-wearing	-	-	-	-	0.64	0.54 - 0.77
Preventive Measures Compliance	-	-	-	-	0.59	0.49 - 0.70

Figure S2. Correlation analysis between risk factors.

A

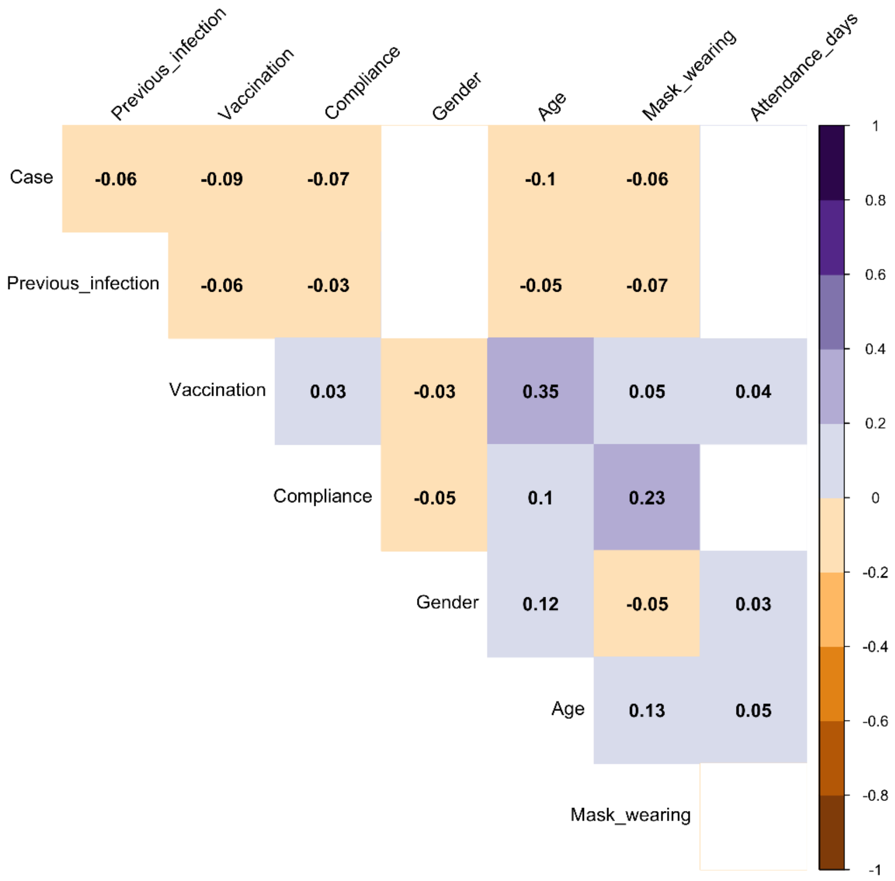

B

**Figure 2b model**	4330	34.98	<.0001
**Same without 'Compliance with protective measures'**	4365		
