# Supplementary material for: Association between two mass-gathering outdoor events and incidence of SARS-CoV-2 infections during the fifth wave of COVID-19 in north-east Spain: A population-based control-matched analysis
Source: Lancet Reg Health Eur. 2022 Feb 28;15:100337. doi: 10.1016/j.lanepe.2022.100337 (PMC8883024; doi:10.1016/j.lanepe.2022.100337)
Supplement: Supplementary file 2 [file mmc2.docx]

**Association between Two Mass-Gathering Outdoor Events and Incidence of SARS-CoV-2 Infections during the Fifth Wave of COVID-19 in North-East Spain: a Population-Based Control-Matched Analysis**

Supplementary material captions

Supplementary materials Captions

**Table S1. Estimate of the number of false negative results according to attendees in each festival, two test sensitivity values, and a range of pre-test probabilities.** The number of false negative (FN) tests was derived from the negative-predictive value (NPV), estimated by considering the prevalence as pre-test probability. Several NPV were modeled for pre-test probabilities ranging between 0.01% to 2% given an expected specificity of 95% and two sensibilities: 70 and 80%. Estimates were modeled separately for the number of attendees in each event and two different sensitivity values.

**Figure S1. Study Profile.** Flow-chart of individual inclusion in the analysis. Flowchart for the first (A) and second (B) festival outdoor music festivals.

**Table S2. Population characteristics.** Demographic characteristics for individuals included in the analysis. SARS-CoV-2 immunity status is specified.

**Table S3. Covid-19 Incidence and relative risk (RR) within different post-event periods**

**Table S4. Odds ratio for COVID-19 infection after attending the events (Numerical values)**. Risk factor multivariate analysis for COVID-19 infection after attending the events. Time frame considered: 3-to-10 days. Definitions: Previous COVID-19 Infection (with or without receiving any vaccine); Full vaccination (i.e., had received the full vaccination regimen >14 days before the event), partial vaccination (i.e., had received an incomplete vaccination regimen or a complete vaccination regimen <14 days before the event); Preventive Measures Compliance and Mask-wearing (OR for answering "all or most of the time"); Age (OR for an increase in one unit); Days of Attendance (OR for each extra day of assistance). These numerical values are represented as a forest plot in Figure 2.

**Figure S2. Correlation analysis between risk factors. (A)** Pearson correlation between risk factors. Coefficients with p values < 0.01 are shown. **(B)** Residual deviance of the logistic regression model including the variables “mask-wearing” and “compliance with the overall preventive measures (Figure 2B of the main text) and excluding the item regarding “compliance with preventive measures” were 4330 and 4365, respectively (F-statistic 34.98; p <.001).
