# Supplementary material for: Association between two mass-gathering outdoor events and incidence of SARS-CoV-2 infections during the fifth wave of COVID-19 in north-east Spain: A population-based control-matched analysis
Source: Lancet Reg Health Eur. 2022 Feb 28;15:100337. doi: 10.1016/j.lanepe.2022.100337 (PMC8883024; doi:10.1016/j.lanepe.2022.100337)
Supplement: Supplementary file 4 [file mmc4.pdf]

CRUÏLLA

Cross-sectional observational study to assess the prevalence of Covid-19 among those attending the Cruïlla festival

Code: SAFEtival

Version 1.0, 27th August 2021

Sponsor:

Cruïlla Barcelona - BARCELONA EVENTS MUSICALS, S.L.
Carrer de Pujades, 77, 2n 7a
08005 Barcelona

Principal investigator:

Oriol Mitjà, MD, PhD

Co-Investigators:

Barbara Baró, PhD
Bonaventura Clotet, MD, PhD
Dan Ouchi, PhD
Miquel Àngel Rodríguez
Clara Suñer, PhD

This document contains confidential information and is disclosed pursuant to confidentiality and non-disclosure obligations. This information should be used solely for the purposes for which it was provided and should not be copied, shared with, or disclosed to any third party without the express written consent of the Sponsor.

ANNEX 3, OP.02.01.FLS-RS.02. Pre-estudi 08/04/2013

1 SUMMARY

1.1 Sponsor identification and Address

Cruïlla Barcelona - BARCELONA EVENTS MUSICALS, S.L.
Carrer de Pujades número 77, 2n 7a
08005 Barcelona

1.2 Title of the study

Cross-sectional observational study to assess the prevalence of Covid-19 among those attending the Cruïlla festival

1.3 Code of the protocol

SAFEtival

1.4 Principal investigator and address

Oriol Mitjà, MD, PhD
Hospital Universitari Germans Trias i Pujol
Ctra. Canyet, s/n
08916 Badalona

1.5 Ethics Committee assessing the protocol

CEIm Hospital Universitari Germans Trias i Pujol
Hospital Universitari Germans Trias i Pujol
Carretera de Canyet s/n
08916 Badalona (Barcelona)

1.6 Primary objective

Prevalence of Covid-19 among those attending the Cruïlla festival.

1.7 Design

Subjects who underwent a SARS-Cov-2 antigen test within the Cruïlla festival where sent an online survey to voluntarily provide data regarding social contacts before the festival, Covid-19 symptoms, and SARS-CoV-2 positive test after the festival.

Data will be transferred anonymously (without any personal identificatory) to investigators from the Germans Trias i Pujol Hospital and Lluita contra la Sida Foundation to be analyzed.

1.8 Study pathology

Covid-19.

1.9 Data from the medicinal product

Not applicable.

1.10 Type of population and number subjects

Study population will be subjects who underwent SARS-Cov-2 antigen test within the Cruïlla festival who answered a voluntary survey after the festival.

Overall, 40000 people underwent SARS-Cov-2 antigen test within the Cruïlla festival. All of them will be sent an online survey, and 13137 provided answers.

1.11 Calendar

Ethics Committee application:	September 2021
Data management:	October 2021
Data analysis:	October 2021
Final report:	October 2021

1.12 Financing source

Cruïlla Barcelona - BARCELONA EVENTS MUSICALS, S.L.
Carrer de Pujades, 77, 2n 7a
08005 Barcelona

ANNEX 3, OP.02.01.FLS-RS.02. Pre-estudi 08/04/2013

2 TABLE OF CONTENTS

1 SUMMARY 2
1.1 Sponsor identification and Address 2
1.2 Title of the study 2
1.3 Code of the protocol 2
1.4 Principal investigator and address 2
1.5 Ethics Committee assessing the protocol 2
1.6 Primary objective 2
1.7 Design 2
1.8 Study pathology 2
1.9 Data from the medicinal product 2
1.10 Type of population and number subjects 2
1.11 Calendar 3
1.12 Financing source 3
2 TABLE OF CONTENTS 4
4. RATIONALE 5
3 OBJECTIVES 7
3.1 Primary objective 7
3.2 Secondary objective 7
4 INFORMATION SOURCE AND FIELD 8
5 STUDY DESIGN 9
5.1 Definition of study population: Selection criteria 9
5.2 Observation period 9
5.3 Treatment and exposure description 9
5.4 Control group selection 9
5.5 Sample size predetermination 9
6 VARIABLES AND MEASURE INSTRUMENTS. MEASUREMENTS DEFINITION AND DESCRIPTION 10
6.1 Primary endpoint 10
6.2 Secondary endpoints 10
6.3 Data collection 10
7 STATISTICAL ANALISIS 12
7.1 Populations for the analyses 12
7.2 Statistical methods 12
8 ETHICAL ASPECTS 13
8.1 Patient information and informed consent 13
8.2 Data protection 13
ANNEX I: SURVEY I – SUBJECTS with negative SARS-CoV-2 antigen result test at Cruïlla screening 15
ANNEX II: SURVEY II – SUBJECTS with positive SARS-CoV-2 antigen result test at Cruïlla screening 15

ANNEX 3, OP.02.01.FLS-RS.02. Pre-estudi 08/04/2013

4. RATIONALE

The importance of the live events industry to the economy is significant. However, the public health response to COVID-19 led to an unprecedented closure in the entertainment industry. Although substantial advances have been made to re-open other industries, there has been limited experience on the reopening of live events such as live music concerts, festivals, or congresses[12]. Because these experiences require people to be very close together, live events generally create conditions that favour virus transmission. Thus, preventive measures need to be adopted. However, there are no clear policy protocols to permit the return of live entertainment at full capacity[34].

Several self-care and risk mitigation strategies are currently being used[32].

1. On the one hand, facemasks wearing, handwashing, and capacity reduction to promote social distancing are non-pharmaceutical interventions available to reduce the basic reproduction rate of the virus.
2. Infection or immunity passports, which refer to tests that demonstrate negative infection status or immunity have also been used to return to activities such as travelling.
3. Finally, given the continued roll out of vaccinations, vaccine passports have become a new criterion to demonstrate immunity in certain activities.

How to combine all these strategies in a cost-efficient manner to celebrate safely live events is still under debate.

On July 8, 9 and 10, 2021, Cruïlla music festival took place in Barcelona, Catalonia, Spain. To reduce the risk of SARS-CoV-2 transmission within the festival, the organizers applied the following measures.

- Attendees to the festival were screened for SARS-CoV-2 infection with Ag-RDT (Rapid Diagnostic Test) via a nasopharyngeal swab.
- Non-contact body temperature screening was performed.
- Attendees were given an official FFP2 (Filtering facepiece 2) mask. The use of the mask was compulsory whenever people were not eating/drinking.

Two weeks after the festival, on July 22, 2021, two anonymous surveys were sent to all attendees, one to individuals who tested negative and accessed the festival (see Annex I) and another one to attendees who tested positive at the festival entrance and were not allowed to enter (see Annex II). This study aims to analyze the answers to these surveys with two main objectives:

1. To assess the prevalence of Covid-19 among those attending the Cruïlla festival in the 10 days following their attendance.
2. To characterize the subjects according to the result of the antigen test performed at the Cruïlla festival and presence/absence of Covid-19 in the 10 days following the last day they attended the festival. This characterization will be specially focused on the demographic characteristics of the attendees and the preventives measures taken at the individual level.

Cruïlla Barcelona has promoted this study in collaboration with Dr. Oriol Mitjà from the Hospital Germans Trias i Pujol and the Fundació Lluita contra la Sida. This analysis will provide information regarding the effectiveness of preventive

ANNEX 3, OP.02.01.FLS-RS.02. Pre-estudi 08/04/2013

measures against COVID-19 transmission taken during the festival, which will be beneficial for the organization of future events.

ANNEX 3, OP.02.01.FLS-RS.02. Pre-estudi 08/04/2013

ANNEX 3, OP.02.01.FLS-RS.02. Pre-estudi 08/04/2013

OBJECTIVES

2.1 Primary objective

Prevalence of Covid-19 among those attending the Cruïlla festival.

2.2 Secondary objective

Characterization of subjects according to the result of the antigen test performed at the Cruïlla festival and presence / absence of Covid-19 in the 10 days following the last day they attended the festival.

3 INFORMATION SOURCE AND FIELD

Data were captured by the Sponsor using the standard quality procedures after the Cruïlla festival.

ANNEX 3, OP.02.01.FLS-RS.02. Pre-estudi 08/04/2013

4 STUDY DESIGN

4.1 Definition of study population: Selection criteria

Study population will be subjects who underwent SARS-Cov-2 antigen test within the Cruïlla festival who answered a voluntary survey after the festival.

4.2 Observation period

Data was collected regarding social contacts 2 days before the Cruïlla festival, and Covid-19 symptoms and/or SARS-CoV-2 positive test within 10 days after the festival.

4.3 Treatment and exposure description

Subjects with negative result of SARS-Cov-2 antigen test were exposed to the Cruïlla festival for 1, 2 or 3 days.

4.4 Control group selection

Prevalence of Covid-19 among those attending the Cruïlla festival will be compared with prevalence of Covid-19 in Catalonia according to the same group characteristics (gender, age, geographical area, and vaccination status), obtained from the Catalan Health System Covid webpage (www.dadescovid.cat) and demographic Catalan data from the Statistical Institute of Catalonia (IdesCat).

4.5 Sample size predetermination

This is an observational descriptive study, with no formal sample size calculation.
Overall, 40000 people underwent SARS-Cov-2 antigen test within the Cruïlla festival. All of them will be sent an online survey, and 13211 provided answers.

ANNEX 3, OP.02.01.FLS-RS.02. Pre-estudi 08/04/2013

5 VARIABLES AND MEASURE INSTRUMENTS. MEASUREMENTS DEFINITION AND DESCRIPTION

5.1 Primary endpoint

To study the prevalence of Covid-19 among those attending the Cruïlla festival, the following endpoints will be analysed:

- Frequency of subjects attending to the Cruïlla festival who report positive laboratory test on SARS-CoV-2 within the 10 days after the festival.

Additional analysis will be performed excluding the following subjects, who will be considered as not infected during the Cruïlla festival:

- Subjects with symptoms before the Cruïlla festival
- Subjects who were in physical close contact (less than 2 meters more than 15 minutes) with known positive-SARS-CoV-2 individuals before the festival.

5.2 Secondary endpoints

The following groups of subjects will be characterized:

- Subjects with positive SARS-CoV-2 antigen result test in the festival screening
- Subjects with negative SARS-CoV-2 antigen result test in the festival screening
- Subjects with negative SARS-CoV-2 antigen result test in the festival screening festival who report positive laboratory test on SARS-CoV-2 within the 10 days after the last day they attended festival.
- Subjects with negative SARS-CoV-2 antigen result test in the festival screening festival who do not report positive laboratory test on SARS-CoV-2 within the 10 days after the last day they attended festival.

For each group, the following characteristics will be described:

- Gender
- Age
- Place of residence
- Covid-19 symptoms
- Level of protection against SARS-CoV-2 infection (high, medium, low - depending on the vaccination status and reinfection)
- Use of mask during the festival
- Number of days of attendance at the festival
- Days of attendance at the festival

5.3 Data collection

Data was collected using two different surveys:

- Subjects with negative SARS-CoV-2 antigen result test in the festival screening answered the "Survey I" (see annex I).

- Subjects with positive SARS-CoV-2 antigen result test in the festival screening answered the "Survey II" (see annex II).

Data collected was self-reported by the study subjects.

ANNEX 3, OP.02.01.FLS-RS.02. Pre-estudi 08/04/2013

In addition to the data collected using the surveys, the following data will be used for the study:

- Aggregated results of the SARS-CoV-2 antigen test performed in the Cruïlla screening of the subjects answering the survey.
- Number of attendants at the festival per day, including age, gender, and place of residence.

ANNEX 3, OP.02.01.FLS-RS.02. Pre-estudi 08/04/2013

6 STATISTICAL ANALISIS

After the validation of the database, its content will be transferred to datasets for statistical analysis. All statistical analyses will be performed using the statistical package R 3.6.3.

6.1 Populations for the analyses

Analysis will be performed using the Full Analysis Set (FAS) population, which will include all subjects who answer the surveys, excluding the following:

- Subjects with positive SARS-CoV-2 antigen result test at Cruïlla screening who answer Survey I.
- Subjects with negative SARS-CoV-2 antigen result test at Cruïlla screening who answer Survey II.

6.2 Statistical methods

All variables will be described for the entire study sample and for each study group. To this end, the following analyses are foreseen:

- Continuous data will be described by their mean, median, standard deviation (SD), quartiles, minimum, and maximum.
- Categorical variables will be described by frequency and percentages (n, %).

Study endpoints will be analysed per groups and using frequencies, as described above in section 7.

No data imputation will be made. Only the subjects observed will be analysed and the number of missing data will be described in each analysis. Answers about contacts with positive people will only be considered if the contact was previous to the positive test or the beginning of the symptoms.

ANNEX 3, OP.02.01.FLS-RS.02. Pre-estudi 08/04/2013

7 ETHICAL ASPECTS

7.1 Patient information and informed consent

Before starting the analysis, the study will be approved by an Independent Ethics Committee (IEC). Individual informed consent will not be obtained, as the database will not contain name, clinic history number or subject initials.

Although this is not an interventional study, it will be designed and implemented according to the ethical principles which have their origin in de Declaration of Helsinki. There are no expected risks associated with the study.

7.2 Data protection

The processing of the data to be compiled will be performed in accordance with Spanish Organic Law 3/2018, which develops the General Data Protection Regulation 2016/679 on data protection and privacy for all individuals within the European Union. The subjects will be guaranteed anonymity.
Attendees were informed about the legal conditions of answering the survey with the following paragraph:

D'acord amb la normativa vigent en l'àmbit de la protecció de dades de caràcter personal, s'informa les persones participants que la complementació del qüestionari és de caràcter voluntari i que les dades que es facilitin durant el mateix es tractaran només a nivell agregat, per la qual cosa es garanteix l'anonimat i el secret estadístic dels participants. En aquest sentit, les dades que es recullin a través d'aquest qüestionari es desaran en un fitxer separat i independent del fitxer on estan registrades les dades personals de les quals ja disposa el Festival Cruïlla, de manera que unes i altres mai es podran associar ni creuar. De cara a aquest projecte, les dades personals de les quals disposa el Festival Cruïlla només es faran servir per a l'enviament del qüestionari i per comunicar a les persones guanyadores del sorteig que han obtingut aquesta condició. En cap cas es faran servir aquestes dades personals durant l'explotació dels resultats recollits a través d'aquest qüestionari.

ANNEX 3, OP.02.01.FLS-RS.02. Pre-estudi 08/04/2013

8 REFERENCES

1 Revollo B, Blanco I, Soler P, *et al.* Same-day SARS-CoV-2 antigen test screening in an indoor mass-gathering live music event: a randomised controlled trial. *Lancet Infect Dis* 2021; **3099**: 1–8.

2 Live DMA website. https://www.live-dma.eu/covid-19/.(accessed August 2021)

3 Centers for Disease Control. Events and gatherings: Guidance for Organizing Large Events and Gatherings. https://www.cdc.gov/coronavirus/2019-ncov/ https://www.cdc.gov/coronavirus/2019-ncov/community/large-events/considerations-for-events-gatherings.html. (accessed August 2021)

4 Harris M, Kreindler J, El-Osta A, Esko T, Majeed A. Safe management of full-capacity live/mass events in COVID-19 will require mathematical, epidemiological and economic modelling. *J R Soc Med* 2021; **114**: 290–4.

ANNEX 3, OP.02.01.FLS-RS.02. Pre-estudi 08/04/2013

ANNEX I: SURVEY I – SUBJECTS with negative SARS-CoV-2 antigen result test at Cruïlla screening

ANNEX II: SURVEY II – SUBJECTS with positive SARS-CoV-2 antigen result test at Cruïlla screening

ANNEX 3, OP.02.01.FLS-RS.02. Pre-estudi 08/04/2013
