# Supplementary material for: Association between two mass-gathering outdoor events and incidence of SARS-CoV-2 infections during the fifth wave of COVID-19 in north-east Spain: A population-based control-matched analysis
Source: Lancet Reg Health Eur. 2022 Feb 28;15:100337. doi: 10.1016/j.lanepe.2022.100337 (PMC8883024; doi:10.1016/j.lanepe.2022.100337)
Supplement: Supplementary file 5 [file mmc5.pdf]

Association between Two Mass-Gathering Outdoor Events and Incidence of SARS-CoV-2 Infections during the Fifth Wave of COVID-19 in North-East Spain

Supplementary Appendix 1. Post-event survey Second Festival

[IDI]

Completa el qüestionari en CATALÀ ***1***

Completa el cuestionario en CASTELLANO ***2***

Complete the questionnaire in ENGLISH ***3***

[ID1]

What is your gender?

Male ***1***
Female ***2***
Non - Binary ***3***

[ID2]

How old are you?

____

[ID3_CP]

What is the postcode of your place of residence?

____

I live outside Spain = 99997

What is your country of residence?
[ID3_PAIS]

Association between Two Mass-Gathering Outdoor Events and Incidence of SARS-CoV-2 Infections during the Fifth Wave of COVID-19 in North-East Spain

Supplementary Appendix 1. Post-event survey Second Festival

***Afghanistan** ... 4*
***Åland Islands** ... 248*
***Albania** ... 8*
***Germany** ... 276*
***Algeria** ... 12*
***Andorra** ... 20*
***Angola** ... 24*
***Anguilla** ... 660*
***Antigua and Barbuda** ... 28*
***Argentina** ... 32*
***Armenia** ... 51*
***Aruba** ... 533*
***Saudi Arabia** ... 682*
***Australia** ... 36*
***Austria** ... 40*
***Azerbaijan** ... 31*
***Bahamas** ... 44*
***Bahrain** ... 48*
***Bangladesh** ... 50*
***Barbados** ... 52*
***Belarus** ... 112*
***Belize** ... 84*
***Benin** ... 204*
***Bermuda** ... 60*
***Bhutan** ... 64*
***Bolivia (Plurinational State of)** ... 68*
***Bonaire, St. Eustatius and Saba** ... 535*
***Botswana** ... 72*
***Brazil** ... 76*
***Brunei Darussalam** ... 96*
***Bulgaria** ... 100*
***Burkina Faso** ... 854*
***Burundi** ... 108*
***Belgium** ... 56*
***Bosnia and Herzegovina** ... 70*
***Cambodia** ... 116*
***Cameroon** ... 120*
***Canada** ... 124*
***Cape Verde** ... 132*
***Colombia** ... 170*
***Comoros** ... 174*
***Congo** [illegible]*

[ID4]

Had you come to the Cruïlla Festival before?

***Yes, I come every year or almost** ... 1*
***Yes, I have come another time** ... 2*
***No, this year is the first time** ... 3*

[P1]

Which day or days did you attend the festival?

***Thursday, July 8** ... 1*
***Friday, July 9** ... 2*
***Saturday, July 10** ... 3*

Association between Two Mass-Gathering Outdoor Events and Incidence of SARS-CoV-2 Infections during the Fifth Wave of COVID-19 in North-East Spain

Supplementary Appendix 1. Post-event survey Second Festival

To what degree do you agree or disagree with the following statements?
[P2]

	Strongly disagree	*Somewhat disagree*	*Somewhat agree*	*Strongly agree*
**I really wanted to attend the festival**	1	2	3	4
**Before I took the antigen test I was worried about the possible result**	1	2	3	4
**I thought the organisation of the antigen tests was adequate**	1	2	3	4
**Taking the antigen test gave me confidence in the event**	1	2	3	4
**The information I received before taking the antigen test was appropriate**	1	2	3	4
**The information I received after taking the antigen test was appropriate**	1	2	3	4
**Finding out the result of the antigen test made me feel good**	1	2	3	4
**The festival attendees in general complied with the stipulated prevention measures against COVID**	1	2	3	4
**It was hard for me to maintain the prevention measures against COVID inside the enclosure**	1	2	3	4

[P3]

Did you get a positive result for any of the antigen tests you took before entering the festival enclosure?

No ***2***
Yes... ***1***

[P3_1]

On what day did you get a positive result?

Thursday, July 8.. ***1***
Friday, July 9... ***2***
Saturday, July 10....................................... ***3***

[P3_2]

Did you take another test afterwards to confirm this result?

Yes, a PCR test and it was positive... ***1***

Yes, a PCR test and it was negative... ***2***

Yes, another antigen test and it was positive... ***3***

Yes, another antigen test and it was negative... ***4***

No, I did no other test afterwards to confirm the result... ***5***

[P4]

While you were in the festival enclosure (including eating, drinking and/or smoking times), did you wear the face covering...

All the time or almost ***1***
Most of the time ***2***
About half the time ***3***
Less than half the time................................. ***4***
Almost never... ***5***

Association between Two Mass-Gathering Outdoor Events and Incidence of SARS-CoV-2 Infections during the Fifth Wave of COVID-19 in North-East Spain

Supplementary Appendix 1. Post-event survey Second Festival

[P5]

We will now ask you some questions about the days before and after the festival.

No ***2***
Yes.. ***1***

Did you have Covid-19 symptoms between the 2 days before you attended the festival and the 10 days afterwards, in other words, between @1@2@3 and @4@5@6 (both days inclusive)?

[P5_1]

On what day did they start?

Tuesday, July 6 .... ***6***
Wednesday, July 7.. ***7***
Thursday, July 8... ***8***
Friday, July 9 .... ***9***
Saturday, July 10... ***10***
Sunday, July 11 .... ***11***
Monday, July 12 .... ***12***
Tuesday, July 13 .... ***13***
Wednesday, July 14...................................... ***14***
Thursday, July 15.. ***15***
Friday, July 16 .... ***16***
Saturday, July 17... ***17***
Sunday, July 18... ***18***
Monday, July 19 .... ***19***
Tuesday, July 20 .... ***20***

[P6]

Did you have a positive Covid-19 PCR or antigen test result on the days after the festival, in other words, between @1@2@3 and @4@5@6 (both days inclusive)?

No ***2***
Yes.. ***1***

[P6_1]

On what day did you take this test?

Friday, July 9 .... ***9***
Saturday, July 10... ***10***
Sunday, July 11 .... ***11***
Monday, July 12 .... ***12***
Tuesday, July 13 .... ***13***
Wednesday, July 14...................................... ***14***
Thursday, July 15.. ***15***
Friday, July 16 .... ***16***
Saturday, July 17... ***17***
Sunday, July 18... ***18***
Monday, July 19 .... ***19***
Tuesday, July 20 .... ***20***

[P7]

Were you in close contact with a person who tested positive for Covid-19 between the 2 days before attending the festival and the 10 days afterwards, in other words, between @1@2@3 and @4@5@6 (both inclusive)?

No ***2***
Yes.. ***1***

[P7_1]

Within this period, what was the first day on which you were in close contact with this person who tested positive?

Tuesday, July 6 .... ***6***
Wednesday, July 7.. ***7***
Thursday, July 8... ***8***
Friday, July 9 .... ***9***
Saturday, July 10... ***10***
Sunday, July 11 .... ***11***
Monday, July 12 .... ***12***
Tuesday, July 13 .... ***13***
Wednesday, July 14...................................... ***14***
Thursday, July 15.. ***15***
Friday, July 16 .... ***16***
Saturday, July 17... ***17***
Sunday, July 18... ***18***
Monday, July 19 .... ***19***
Tuesday, July 20 .... ***20***

Association between Two Mass-Gathering Outdoor Events and Incidence of SARS-CoV-2 Infections during the Fifth Wave of COVID-19 in North-East Spain

Supplementary Appendix 1. Post-event survey Second Festival

[P7_2]

Did this close contact take place in the festival enclosure while it was being held?

***No** 2*
***Yes**.. 1*

[P7_3]

Did this person with whom you were in close contact know they were Covid-19 positive at the test conducted by the Cruïlla Festival?

***No** 2*
***Yes**.. 1*

[P8]

Before the festival had you ever tested positive for Covid-19 from a PCR or an antigen test?

***No** 2*
***Yes**.. 1*

[P8_1]

From what date approximately is this test?

***Early July 2021** 1*
***June 2021**... 2*
***May 2021** 3*
***April 2021**... 4*
***March 2021** 5*
***February 2021**.. 6*
***January 2021** 7*
***December 2020**.. 8*
***November 2020** 9*
***October 2020** 10*
***September 2020** 11*
***August 2020**... 12*
***July 2020**.. 13*
***June 2020**... 14*
***May 2020** 15*
***April 2020**... 16*
***March 2020** 17*
***February 2020**.. 18*
***January 2020** 19*

[P9]

Before the festival had you been vaccinated against Covid-19?

***No** 1*
***Half vaccinated (I was waiting for the second dose)**... 2*
***Yes, fully vaccinated**... 3*

[P9_1]

Which vaccine were you given as the first dose?

***Pfizer**... 1*
***Moderna**... 2*
***AstraZeneca** 3*
***Don't know</span** 98*

[P9_2]

When were you given the first dose?

***Early July 2021** 1*
***June 2021**... 2*
***May 2021**.. 3*
***April 2021**... 4*
***March 2021** 5*
***February 2021**.. 6*
***January 2021** 7*
***December 2020**.. 8*

[P9_3]

Which vaccine were you given as the last dose?

***Pfizer**... 1*
***Moderna**... 2*
***AstraZeneca** 3*
***Janssen**.. 4*
***Don't know</span** 98*

Supplementary Appendix 1. Post-event survey Second Festival

[P9_4]

When were you given the last dose?

***Early July 2021** 1*
***June 2021**... 2*
***May 2021** 3*
***April 2021**... 4*
***March 2021** 5*
***February 2021**.. 6*
***January 2021** 7*
***December 2020**.. 8*

To which degree do you agree or disagree with the following statements on the festival?

[P10]

	Strongly disa-gree	*Somewhat disa-gree*	*Somewhat agree*	*Strongly agree*
**I felt safe in the face of the pandemic while I was in the enclosure**	1	2	3	4
**There was a moment when I was scared of getting infected**	1	2	3	4
**I thought the protective measures against COVID implemented by the organisation were adequate**	1	2	3	4
**I was able to enjoy the festival**	1	2	3	4
**I am satisfied with the organisation of the festival**	1	2	3	4
**I would like to attend the festival again next year**	1	2	3	4
**I feel that by attending the festival I was helping with the research to hold safe events during the pandemic**	1	2	3	4

[OBS]

We leave you this space in case you want to add any comments.

[FI_ENQ]
