# Supplementary material for: Association between two mass-gathering outdoor events and incidence of SARS-CoV-2 infections during the fifth wave of COVID-19 in north-east Spain: A population-based control-matched analysis
Source: Lancet Reg Health Eur. 2022 Feb 28;15:100337. doi: 10.1016/j.lanepe.2022.100337 (PMC8883024; doi:10.1016/j.lanepe.2022.100337)
Supplement: Supplementary file 6 [file mmc6.pdf]

Cross-sectional observational study to assess the prevalence of Covid-19 among those attending a music festival using the Public Health registries

Code: SAFEtival_Salut

Version 2.0, 22nd October 2021

Sponsor:
Conselleria de Salut
Generalitat de Catalunya
Pavelló Ave Maria, Travessera de les Corts, 131, 159,
08028 Barcelona

Principal investigator:

Oriol Mitjà, MD, PhD

Co-Investigators:

Barbara Baro, PhD
Bonaventura Clotet, MD, PhD
Ermengol Coma, MD
Manuel Medina, PhD
Dan Ouchi, PhD
Miquel Àngel Rodríguez
Clara Suñer, PhD

This document contains confidential information and is disclosed pursuant to confidentiality and non-disclosure obligations. This information should be used solely for the purposes for which it was provided and should not be copied, shared with, or disclosed to any third party without the express written consent of the Sponsor.

1 SUMMARY

1.1 Sponsor identification and Address

Conselleria de Salut
Generalitat de Catalunya
Pavelló Ave Maria, Travessera de les Corts, 131, 159,
08028 Barcelona

1.2 Title of the study

Cross-sectional observational study to assess the prevalence of Covid-19 among those attending a music festival using the Public Health registries

1.3 Code of the protocol

SAFEtival_Salut

1.4 Principal investigator and address

Oriol Mitjà, MD, PhD
Hospital Universitari Germans Trias i Pujol
Ctra. Canyet, s/n
08916 Badalona

1.5 Ethics Committee assessing the protocol

CEIm Hospital Universitari Germans Trias i Pujol
Hospital Universitari Germans Trias i Pujol
Carretera de Canyet s/n
08916 Badalona (Barcelona)

1.6 Primary objective

Prevalence of Covid-19 among those attending the Cruïlla festival and the CanetRock festival.

1.7 Design

This is a retrospective cohort study comparing the incidence of Covid-19 between individuals that attended the Cruïlla music festival and a control cohort that did not attend this event. It will also compare the incidence of Covid-19 between individuals that attended the CanetRock music festival and its corresponding control cohort that did not attend this event.

The attendees and the control cohort will be paired according to the following characteristics: age, gender, geographical area and COVID-19 immune status (vaccine/reinfection). The study will compare the number of individuals who tested positive for Covid-19 in each cohort, before and after the festival.

Briefly, data identifying Cruïlla festival attendees and CanetRock festival attendees will be transferred to the Catalan Health Authorities, who will collect information regarding their Covid-19 vaccination status from the Public Health registries. Each attendee will be paired to a control subject (based on age, gender, geographical area and Covid-19 immune status). Then, the number of positive SARS-CoV-2 tests between attendees and

controls on the 14 days after the festival will be compared. Data regarding positive SARS-Cov-2 tests after the festival of the attendees and the control group will be collected from the Catalan Health Department registry.

This data will be transferred anonymously (without any personal identificatory) to investigators from the Germans Trias i Pujol Hospital and Lluita contra la Sida Foundation to be interpreted.

1.8 Study pathology

COVID-19.

1.9 Data from the medicinal product

Not applicable.

1.10 Type of population and number subjects

Study population will be subjects who underwent SARS-Cov-2 antigen test within the Cruïlla festival or the CanetRock Festival, and who are registered at the Catalan Public Health System. Overall, 40000 people underwent SARS-Cov-2 antigen test within the Cruïlla festival. For the CanetRock Festival, 22000 people underwent SARS-CoV-2 antigen test before attending the festival. The study will also include data from a control cohort, made of subjects that did not attend the event but were paired with the attendees. Pairing will be made 1:1 based on age, gender, geographical area and Covid-19 immune status (vaccine/previous infection).

1.11 Calendar

Ethics Committee application:	October 2021
Data management:	November 2021
Data analysis:	November 2021
Final report:	November 2021

1.12 Financing source

Internal research funding sources within both partners, FLS and Heath Ministry of *Generalitat de Catalunya*.

2 TABLE OF CONTENTS

1 SUMMARY 2
1.1 Sponsor identification and Address 2
1.2 Title of the study 2
1.3 Code of the protocol 2
1.4 Principal investigator and address 2
1.5 Ethics Committee assessing the protocol 2
1.6 Primary objective 2
1.7 Design 2
1.8 Study pathology 3
1.9 Data from the medicinal product 3
1.10 Type of population and number subjects 3
1.11 Calendar 3
1.12 Financing source 3
2 TABLE OF CONTENTS 4
3 RATIONALE 5
4 OBJECTIVES 7
4.1 Primary objective 7
4.2 Secondary objective 7
5 INFORMATION SOURCE AND FIELD 8
6 STUDY DESIGN 9
6.1 Definition of study population: Selection criteria 9
6.2 Observation period 9
6.3 Treatment and exposure description 9
6.4 Control group selection 9
6.5 Sample size predetermination 9
7 VARIABLES AND MEASURE INSTRUMENTS. MEASUREMENTS DEFINITION AND DESCRIPTION 10
7.1 Primary endpoint 10
7.2 Secondary endpoints 10
7.3 Data collection 10
8 STATISTICAL ANALISIS 12
8.1 Populations for the analyses 12
8.2 Statistical methods 12
9 ETHICAL ASPECTS 13
9.1 Ethical considerations 13
9.2 Subject’s consent and data protection 13
10 REFERENCES 15

3 RATIONALE

The importance of the live events industry to the economy is significant. However, the public health response to COVID-19 led to an unprecedented closure in the entertainment industry. Although substantial advances have been made to re-open other industries, there has been limited experience on the reopening of live events such as live music concerts, festivals, or congresses[12]. Because these experiences require people to be very close together, live events generally create conditions that favour virus transmission. Thus, preventive measures need to be adopted. However, there are no clear policy protocols to permit the return of live entertainment at full capacity[34].

Several self-care and risk mitigation strategies are currently being used[32].
On the one hand, facemasks wearing, handwashing, and capacity reduction to promote social distancing are non-pharmaceutical interventions available to reduce the basic reproduction rate of the virus.
Infection or immunity passports, which refer to tests that demonstrate negative infection status or immunity have also been used to re-open activities such as travelling.
Finally, given the continued roll out of vaccinations, vaccine passports have become a new criterion to demonstrate immunity in certain activities.
How to combine all these strategies in a cost-efficient manner to celebrate safely live events is still under debate.

On July 3, 2021, CanetRock music festival took place in Canet, a town located 40 km north-east of Barcelona, Catalonia, Spain. On July 8, 9 and 10, 2021, Cruïlla music festival took place in Barcelona, Catalonia, Spain. To reduce the risk of SARS-CoV-2 transmission within the festival, the organizers applied the following measures.

- Attendees to the festival were screened for SARS-CoV-2 infection with Antigen Rapid Diagnostic Test (Ag-RDT) via a nasopharyngeal swab.
- Non-contact body temperature screening was performed.
- Attendees were given a certified Filtering facepiece 2 (FFP2) mask. The use of the mask was compulsory whenever people were not eating/drinking.

This study aims to compare the prevalence of COVID-19 among those attending the Cruïlla festival and a control cohort, before and after attending the festival. Independently, it will also compare the prevalence of COVID-19 among those attending the CanetRock festival and its corresponding control cohort, before and after attending the festival. We will also characterize the subjects developing or not COVID-19 within 14 days after attending the festival. This characterization will be specially focused on the socio-demographic characteristics of the attendees (age, gender, geographic location), the individual level of COVID-19 immunity (based on vaccination and/or previous infections) and the behaviour at the festival (number of days attended, and days of attendance).

Catalan Health Department (*Conselleria de Salut, Generalitat de Catalunya*) has sponsored this study in collaboration with Dr. Oriol Mitjà from the *Hospital Germans Trias i Pujol* and the *Fundació Lluita contra la Sida*, the Cruïlla Barcelona Festival and the CanetRock Festival. This analysis will provide information regarding the

effectiveness of preventive measures against COVID-19 transmission taken during the festivals, which will be beneficial for the organization of future events.

4 OBJECTIVES

4.1 Primary objective

Prevalence of Covid-19 among those attending the Cruïlla Music festival 2021 and the CanetRock music festival 2021.

4.2 Secondary objective

Characterization of subjects according to development or not of COVID-19 within 14 days after attending the festival.

5 INFORMATION SOURCE AND FIELD

Information source will be data captured by Cruïlla festival and CanetRock festival and by the Catalan Health Deparmtent using standard procedures for their activities (see section 7.1 Data Protection for details).

6 STUDY DESIGN

6.1 Definition of study population: Selection criteria

Study population will be subjects who underwent SARS-Cov-2 antigen test within the Cruïlla festival, and a control cohort of paired subjects according to the following characteristics of the attendees (age, gender, geographical area, Covid-19 immune status (vaccine/previous infection). In parallel, a second study population will comprise subjects who underwent SARS-Cov-2 antigen test within the CanetRock festival, and a control cohort of paired subjects according to the previously specified criterion.

6.2 Observation period

Data regarding the number of SARS-CoV-2 positive test results in each cohort will be collected considering 14 days before and 14 days after the festival (24th June - 24th July).

6.3 Treatment and exposure description

For the Cruïlla Festival cohort, subjects with negative results at the SARS-Cov-2 Ag-RDT screening were exposed to the Cruïlla festival for 1, 2 or 3 days.
For the CanetRock Festival cohort, subjects with negative results at the SARS-Cov-2 Ag-RDT screening were exposed to the CanetRock festival for 1 day.

6.4 Control group selection

Prevalence of COVID-19 among those attending the Cruïlla festival or the CanetRock festival will be compared with the prevalence of COVID-19 in a control cohort. The control cohort will be created according to the characteristics of the attendees (gender, age, geographical area, and COVID-19 immune status).

6.5 Sample size predetermination

This is a retrospective cohort study, with no formal sample size calculation. Overall, 40000 people underwent SARS-CoV-2 antigen test within the Cruïlla festival and 22000 people underwent SARS-CoV-2 antigen test within the CanetRock festival. Those that are registered at the Catalan Health System will be paired with a control and included in the analysis.

7 VARIABLES AND MEASURE INSTRUMENTS. MEASUREMENTS DEFINITION AND DESCRIPTION

7.1 Primary endpoint

To study the prevalence of Covid-19 among those attending the festivals, the following endpoint will be analysed:

- Frequency of subjects attending to the Cruïlla festival who report a positive test on SARS-CoV-2 within the 14 days after the festival.
- Frequency of subjects attending to the CanetRock festival who report a positive test on SARS-CoV-2 within the 14 days after the festival.
- Frequency of subjects in each control cohort who report a positive test on SARS-CoV-2 within the 14 days after the festival.

Additional analysis will be performed excluding the following subjects, who will be considered as not infected during the festivals:

- Subjects who tested positive on SARS-CoV-2 6 days after their last day attending the festival.
- Subjects who tested positive within 2 days after attending the festival.

7.2 Secondary endpoints

The following groups of subjects will be characterized:

- Subjects with positive SARS-CoV-2 antigen result test at the Ag-RDT screening
- Subjects with negative SARS-CoV-2 antigen result test at the Ag-RDT screening
- Subjects with negative SARS-CoV-2 antigen result test at the Ag-RDT screening with a positive test on SARS-CoV-2 after attending the festival.
- Subjects with negative SARS-CoV-2 antigen result test at the Ag-RDT screening without a positive test on SARS-CoV-2 after attending the festival.

For each group, the following characteristics will be described:

- Gender
- Age
- Place of residence
- Vaccination level (vaccine received, number of doses, date of vaccination)
- Reinfection status (date of previous SARS-CoV-2 infection, if any)
- Number of days attending the festival
- Days of attendance to the festival

7.3 Data collection

Ag-RDT result, and demographics, of the attendees was collected by Cruïlla Festival and the CanetRock Festival, after the individuals agreed to data capture for epidemiological and research purposes.

Data regarding vaccination status, and positive SARS-Cov-2 test of the attendees at the festival and the control group will be collected from the Catalan Health Department registry.

In addition to the data about the subjects who attended the festival, the following data will be used for the study:

- Number of subjects that purchased an entrance, who tested positive for SARS-CoV-2 before the festivals and returned their entrance (without participating in the Ag-RDT screenings nor attending the festivals).
- Aggregated results of SARS-CoV-2 tests for the control cohort, for the complete cohort and subdivided according to their COVID-19 immunity status.

8 STATISTICAL ANALISIS

After the validation of the database, its content will be transferred to datasets for statistical analysis. All statistical analyses will be performed using the statistical package R 3.6.3.

8.1 Populations for the analyses

Analysis will be performed using the Full Analysis Set (FAS) population, which will include all subjects who underwent SARS-Cov-2 antigen test within the Cruïlla festival. An independent analysis will be done using again the Full Analysis Set (FAS) population, including all subjects who underwent SARS-Cov-2 antigen test within the CanetRock festival. Those for whom information about their Covid-19 vaccination status is available at the Catalan Health registry will be paired with a control subject and included in the analysis.

8.2 Statistical methods

All variables will be described for the entire study sample and for each study group. To this end, the following analyses are foreseen:

- Continuous data will be described by their mean, median, standard deviation (SD), quartiles, minimum, and maximum.
- Categorical variables will be described by frequency and percentages (n, %).

Study endpoints will be analysed per groups and using frequencies, as described above in section 7.

No data imputation will be made. Only the subjects observed will be analysed and the number of missing data will be described in each analysis.

9 ETHICAL ASPECTS

9.1 Ethical considerations

The study will be carried out in strict compliance with the ethical principles of clinical research and the legislation in force in Spain. The study will be approved by an Independent Ethics Committee (IEC).

Although this is not an interventional study, it will be conducted in accordance with national and international standards (Declaration of Helsinki, last update Fortaleza, Brazil 2013) on ethical issues.

9.2 Subject's consent and data protection

The study will comply with the provisions of Regulation (EU) 2016/679 of the European Parliament and of the European Council of 27 April on Data Protection (RGPD), and Organic Law 3/2018, of 5 December on Data Protection personal and guarantee of digital rights (BOE of 6 December 2018).

This is a study without intervention and does not pose any risk or inconvenience to patients. All the data used in this study is provided by the health provider and institutions of the *Generalitat de Catalunya* for the provision and surveillance of public health services and for the management of the COVID-19 pandemic. The procedure for pseudonymizing the data is done at source and is already anonymized for all the analyses carried out by the research team. Due to the characteristics of the project, which obtains data from existing routine records and processes the data for scientific research purposes and to answer a question of public interest, it is considered that there is no need to request consent to subjects.

Cruïlla Festival informed the attendees about the legal conditions of purchasing the entrance and participating in the Ag-RDT screening with the following paragraph:

> *Los datos de carácter personal facilitados van a pasar a formar parte de un fichero, cuyo responsable es BARCELONA EVENTS MUSICALS SL., con CIF nº B65505257 y domicilio en C/Pujades 77, 2 7, dirección de correo electrónico festival@cruillabarcelona.com. Los mencionados datos se utilizarán para la tramitación del proceso descrito. Es necesario facilitarlos con el fin de poder realizar el test y acceder al evento. Los datos proporcionados se conservarán durante todo el tiempo que en que sean necesarios para los fines para los que fueron recabados, así como durante todos los años en que sea preciso para dar cumplimiento a las obligaciones legales. Estos datos solo serán objeto de cesión a la Conselleria de Salut de la Generalitat de Catalunya para el estudio y la investigación. Según lo establecido en la Ley de Protección de datos de carácter personal, usted tiene derecho a acceder a los referidos datos, rectificar aquellos que sean inexactos y solicitar su cancelación o supresión cuando no sean necesarios para los fines para los que fueron recabados, así como, en su caso, a ejercitar los derechos de oposición o portabilidad. Para ejercitar cualquiera de estos derechos deberá presentar un escrito en la siguiente dirección festival@cruillabarcelona.com. En caso de considerar vulnerado su derecho a la protección de datos personales, podrá interponer una reclamación ante la Agencia Española de Protección de Datos (www.agpd.es).*

CanetRock Festival informed the attendees about the legal conditions of purchasing the entrance and participating in the Ag-RDT screening with the following paragraph:

> *Les dades facilitades pels assistents seran custodiades per l'organització i podran ser posades a disposició de les autoritats sanitàries, si són requerides per aquestes, durant almenys un mes amb posterioritat a la celebració del festival. La recollida i custòdia de les dades dels assistents en matèria de salut es farà d'acord amb el que estableix la normativa en matèria de protecció de dades de caràcter personal, i amb la finalitat exclusiva de ser cedides, únicament, a les autoritats sanitàries. Transcorregut un mes des de la recollida de les dades n'hauran de ser suprimides d'acord amb el que estableix la normativa en matèria de protecció de dades de caràcter personal.*

According to this, Cruïlla Festival and CanetRock Festival sent the data from the Ag-RDT screening to the Catalan Health Authorities (within one month from the festival date). At the Catalan Public Health Institute, an independent department from the research team will perform the pseudonymisation of the data, and merge the vaccination status and Covid-19 diagnostic results of the pseudonymised attendees to the festival and the control cohort according to their standardized procedures, which are explained below. The resulting pseudonymized database will be transferred to the research team.

Anonymization procedure

All study subjects will be assigned an anonymized code making impossible for the research team to identify participants. The different databases will be linked through this code which is created by a specific algorithm of automatic coding from the CIP by members that are not part of the research team. Databases with this code are regularly dumped in an official environment created ad-hoc by healthcare institutions in order to monitor the COVID-19 epidemic. Under no circumstances will the code be decoded. Patient confidentiality rules will be met throughout the project. Under no circumstances personal data will be used.

10 REFERENCES

1 Revollo B, Blanco I, Soler P, *et al.* Same-day SARS-CoV-2 antigen test screening in an indoor mass-gathering live music event: a randomised controlled trial. *Lancet Infect Dis* 2021; **3099**: 1–8.

2 Live DMA website. https://www.live-dma.eu/covid-19/.(accessed August 2021)

3 Centers for Disease Control. Events and gatherings: Guidance for Organizing Large Events and Gatherings. https://www.cdc.gov/coronavirus/2019-ncov/ https://www.cdc.gov/coronavirus/2019-ncov/community/large-events/considerations-for-events-gatherings.html. (accessed August 2021)

4 Harris M, Kreindler J, El-Osta A, Esko T, Majeed A. Safe management of full-capacity live/mass events in COVID-19 will require mathematical, epidemiological and economic modelling. *J R Soc Med* 2021; **114**: 290–4.
